# Estradiol enhances thermoregulation induced by ostruthin, a TREK channel agonist, in ovariectomized rats

**DOI:** 10.1016/j.jphyss.2025.100044

**Published:** 2025-09-26

**Authors:** Yuki Uchida, Shotaro Kamijo, Yuki Samejima, Hiroshi Onimaru, Masahiro Hosonuma, Hikaru Isobe, Keiko Ikeda, Motoyasu Honma, Yuri Masaoka, Masahiko Izumizaki

**Affiliations:** a Department of Physiology, Showa Medical University School of Medicine, Tokyo, Japan; b Division of Physiology, Toxicology and Therapeutics, Department of Pharmacology, Showa Medical University, Tokyo, Japan; c Department of Orthopaedic Surgery, Showa Medical University Fujigaoka Hospital, Yokohama, Japan; d Division of Medical Pharmacology, Department of Pharmacology, Showa Medical University School of Medicine, Tokyo, Japan; e Department of Oral Physiology, Showa Medical University School of Dentistry, Tokyo, Japan

**Keywords:** Ostruthin, Estradiol, TREK, Thermoregulation, Menopause

## Abstract

Menopausal women frequently report contradictory thermoregulatory symptoms (hot flashes and chills), believed to result from declining estradiol (E₂) levels; however, mechanisms remain unclear. TWIK-related (TREK) potassium channels function as cold receptors. Although E₂ enhances TREK1 activity *in vitro*, its effect on TREK-mediated thermoregulation has not been investigated *in vivo*. This study investigated whether E₂ facilitated TREK-mediated thermoregulation in ovariectomized rats using ostruthin, a TREK agonist. Rats were ovariectomized and implanted with silastic tubes with or without E₂, followed by ostruthin or vehicle injection. We measured thermoregulatory parameters, plasma hormones (triiodothyronine and thyroxine), and mRNA expression of cold receptors in dorsal root ganglia. Ventral root responses were examined *in vitro*. Ostruthin increased body temperature in E₂(+) versus E₂(−) groups, with increased triiodothyronine and upregulation of *Trek1*, *Vgf*, and *Nos1*. Ostruthin enhanced ventral root responses. These findings demonstrate that E₂ potentiates TREK-mediated thermoregulation through enhanced cold sensing, providing insights into menopausal disorders.

## Highlights

• E_2_ enhances TREK-mediated thermoregulation in a menopausal rat model.
• E_2_ upregulates TREK1 expression and facilitates peripheral cold sensations.
• These findings provide novel insights into the mechanisms of thermoregulatory disorders in menopause.

## Introduction

Body temperature (T_b_) is maintained within a narrow range through autonomic responses that control heat production and dissipation, as well as thermoregulatory behavior. For example, cold exposure triggers responses that conserve and generate heat, including vasoconstriction, shivering, and non-shivering thermogenesis, along with behavioral responses such as cold-escape behavior. These physiological thermoregulatory responses are modulated by factors such as estrogen [1]. The decline in estrogen during menopause becomes problematic because it causes the estrogen-mediated thermoregulatory function identified in this study to cease functioning. A survey has revealed two distinct yet seemingly contradictory thermoregulatory symptoms that are frequently reported by menopausal women. Hot flashes, affecting 3.0–22.1 % of this population, are characterized by excessive heat dissipation through peripheral vasodilation and sweating. In contrast, chilliness, affecting 29.3 % of the population, is associated with vasoconstriction and insufficient heat distribution in peripheral regions [2]. While these symptoms represent opposite vascular responses and thermal imbalances, they are likely attributable to the abrupt decline in estrogen signaling during menopause due to decreased levels of estradiol (E_2_), the most potent form of estrogen in women of reproductive age [3]. However, the precise mechanisms of estrogen action on thermoregulation and how estrogen deficiency leads to such dysregulation remain to be fully elucidated. These knowledge gaps limit the development of targeted therapeutic interventions that can improve the quality of life of menopausal women experiencing thermoregulatory symptoms.

Therefore, we have investigated how E_2_ modulates autonomic and behavioral thermoregulatory responses during cold exposure. Using ovariectomized rats as a menopausal model exposed to cold, we showed that E_2_ enhances autonomic thermoregulation via the medial preoptic area of the hypothalamus by promoting the vasoconstriction-mediated suppression of heat loss [4] from the tail [5], [6] and that systemic E_2_ administration enhances behavioral thermoregulation in the same menopausal model by accelerating tail-hiding behavior, in which the rats tuck their tails under their bodies to reduce heat dissipation [7]. These results demonstrate that E_2_ maintains T_b_ under cold conditions by modulating autonomic and behavioral thermoregulatory mechanisms.

Recently, we investigated the mechanism by which E_2_ modulates peripheral thermal sensing. Thermoregulation involves coordinated actions between the peripheral thermosensors that detect ambient and somatic thermal signals, as well as central integration centers in the brain. These afferent signals are transmitted to the hypothalamus, which processes information and activates the appropriate autonomic and behavioral responses. At the peripheral level, thermal signals are primarily detected by specific thermosensitive ion channels expressed in the sensory neurons [8]. Transient receptor potential (TRP) channels are key molecular thermosensors with different family members activated at distinct temperature ranges [9]. We investigated the effect of E_2_ on thermoregulation via two cold receptors: TRPM8 and TRPA1. E_2_ attenuates the increase in T_b_ induced by L-menthol, a TRPM8 agonist, in ovariectomized rats under thermoneutral conditions [10]. In contrast, E_2_ did not affect the reduction of tail skin temperature (T_tail_) induced by cinnamaldehyde, a TRPA1 agonist, in ovariectomized rats under similar conditions [11]. These findings indicate that E_2_ modulates thermal signaling in a receptor-specific manner at the level of the peripheral thermosensitive ion channels.

Although TRP channels are well-established thermosensors, they work cooperatively with another family of ion channels, the TWIK-related (TREK) potassium channels, to regulate thermal responses [12]. TREK1 and TREK2 are expressed in the skin sensory neurons [12] and modulate neuronal excitability in response to temperature changes within distinct physiological ranges [13]. *In vitro* studies have demonstrated that TREK1 is activated between 14°C and 40°C [14], while TREK2 responds to moderate ambient cool temperatures between 20°C and 25°C, although it can also be activated at warmer temperatures between 40°C and 46°C [15]. *In vivo*, *Trek2-/-* mice showed a decreased latency in tail immersion tests at mildly cold temperatures [15]. Despite their responsiveness across temperature ranges, both channels were activated at cool temperatures, suggesting their role in cold reception.

The potential role of TREK channels in E_2_-mediated thermoregulation has emerged as a promising research topic. *In vitro* electrophysiological studies have demonstrated that E_2_ enhances TREK1 activation in HEK cells [16], suggesting a molecular link between E_2_ and TREK1, though thermoregulatory relevance remains to be determined. Our recent demonstration that ostruthin, a TREK1 and TREK2 agonist [17], attenuates the gradual decline in T_b_ under thermoneutral conditions in ovariectomized rats lacking estrogen supplementation, a commonly used menopausal model [18], indicates that TREK channel activation itself can influence thermoregulation in the absence of E_2_. These findings suggest that TREK channels may represent an important mechanism underlying thermoregulatory responses influenced by E_2_. However, how changes in the E_2_ status during menopause affect TREK-mediated thermoregulation and contribute to menopausal thermoregulatory dysfunction remains unclear.

Based on the similar thermoregulatory effects of E_2_ and TREK channel activation, we hypothesized that E_2_ facilitates thermoregulation, at least in part, by enhancing TREK channel function. This hypothesis was supported by: (i) *in vitro* evidence of TREK1 activation by E_2_ and (ii) comparable *in vivo* effects of E_2_ and TREK agonists. To test this hypothesis, we examined whether E_2_ facilitated TREK-mediated thermoregulation in ovariectomized rats using ostruthin, a TREK1 and TREK2 agonist. In this study, TREK-mediated thermoregulation refers to T_b_ regulation involving TREK channels, primarily through their effects on thermal sensing, and downstream autonomic and behavioral responses. We propose that the disruption of the E_2_–TREK interaction during menopause may impair temperature sensing and thermoregulatory output, potentially explaining the coexistence of hot flashes and chilliness. We assessed this interaction using an integrated approach to analyze thermal regulation across behavioral, autonomic, molecular, and electrophysiological domains.

## Materials and methods

### Animals

Sixty-eight female virgin Wistar rats (body weight 158 ± 0.3 g; age, 9 weeks; Japan SLC, Hamamatsu, Japan) were used for *in vivo* studies for heat loss, thermoregulatory behavior, and metabolic parameter measurements. Thirteen 0- to 4-day-old Wistar rats (either sex; Japan SLC) were used for *in vitro* studies. The rats for *in vivo* studies were individually housed in cages (37 × 21 × 19 cm) at an ambient temperature of 23 ± 1°C with a 12:12-h light-dark cycle; lights were on at 08:00. The rats had ad libitum access to food and water. All experimental protocols were approved by the Animal Research Committee of Showa Medical University (Tokyo, Japan) (approval number: 03110).

### Surgical preparations: data logger implantation, ovariectomy, and E_2_ administration for measurements

All surgical procedures were performed one week before the experiment. This study focused on the interaction between E_2_ and TREK channels in a menopausal model. Therefore, we included only ovariectomized rats with or without E_2_ supplementation. This design allowed us to directly examine the effects of E_2_ on TREK-mediated thermoregulation under controlled hormonal conditions, which was the primary aim of our study. All surgical procedures, including ovariectomies, were performed under isoflurane inhalation anesthesia (Pfizer Japan, Tokyo, Japan). First, following a medial skin incision, a built-in data logger equipped with a temperature sensor and triaxial accelerometer as an activity sensor (nano tag®, KISSEI COMTEC CO., LTD., Matsumoto, Japan) was implanted into the peritoneal cavity of each rat. The data logger measured T_b_ with an accuracy of ±0.5°C and locomotor activity (Act) for thermogenesis through muscle activation, with Act detected by the triaxial accelerometer as frequency.

Subsequently, bilateral ovariectomy was performed through a dorsal skin incision, and a silastic tube (Dow Corning Toray Co., Ltd, Tokyo, Japan) containing crystalline 17ß-estradiol ((E_2_(+), 22.3 mg); Sigma-Aldrich, St. Louis, MO, USA) or an empty tube (E_2_(–)) was placed beneath the right dorsal skin. E_2_ administration began immediately after tube implantation, with the hormone diffusing continuously through the silastic membrane throughout the one week between the surgery and experimentation. This subcutaneous silastic tube method allows for the controlled, sustained release of E_2_ through diffusion across the silastic membrane, providing stable hormone levels over the experimental period [19], [20]. To prevent postoperative infection, the animals received a subcutaneous injection of penicillin G (1000 U; Meiji Pharmaceutical, Tokyo, Japan). By our Institutional Animal Care Committee guidelines and approved protocol (protocol number: 03110), no additional analgesics were administered beyond the anesthesia used during surgery. Using established intervention criteria, the animals were closely monitored for signs of distress throughout the recovery period. The surgical procedures and methods for E_2_ administration were described in our previous studies [10], [21].

After surgery, plasma E_2_ levels were expected to follow the patterns observed in our previous studies using identical surgical procedures and E_2_ administration methods [7], [10], [11]. In those past studies, we confirmed that E_2_(+) rats maintained plasma E_2_ levels comparable to those observed during the proestrus phase of the estrous cycle in rats [22], whereas E_2_(–) rats showed significantly decreased levels one week after ovariectomy. In our previous study [23], we demonstrated that the E_2_ levels in E_2_(–) rats were significantly lower than those in sham-operated controls, confirming the effectiveness of our ovariectomy model for inducing E_2_ depletion. While we did not directly measure E_2_ levels in the current study, the one-week interval between surgery and experiments allowed for postsurgical recovery and stabilization of plasma E_2_ levels based on our well-established protocol.

### Heat loss and thermoregulatory behavior measurements

We measured heat loss responses and thermoregulatory behaviors in ovariectomized rats to examine the effects of the TREK agonist and E_2_ on thermoregulation. Each E_2_(+) and E_2_(−) group was further divided into Vehicle and TREK agonist subgroups, resulting in four experimental groups: Vehicle/E_2_(–), Vehicle/E_2_(+), TREK agonist/E_2_(–), and TREK agonist/E_2_(+). At 18:00 on the day before the experiment (6 d after surgery), each rat was placed in a polyethylene box (19.8 × 30.3 × 48.5 cm), which was then moved to a climatic chamber (Program Incubator IN804, Yamato Scientific, Tokyo, Japan) maintained at 27°C with a 12:12-h light-dark cycle; lights were on at 08:00. The rats had free access to food and water until 08:00 on the experimental day, when the food and water were removed. During the experimental measurements (8:00–12:00), the rats did not have access to food or water. The T_b_ and Act were measured at 08:00 on the experimental day (7 d after surgery). Data collected between 9:30 and 10:00 were used as the baseline values. At 10:00, each rat was removed from the chamber and immediately administered an intraperitoneal injection of either vehicle (0.002 % dimethyl sulfoxide [DMSO; FUJIFILM Wako Pure Chemical Corporation, Osaka, Japan] in phosphate-buffered saline [PBS], total volume 200 μL) or ostruthin (TREK1 and TREK2 agonist; Toronto Research Chemicals, Toronto, ON, Canada; 4.2 μg dissolved in 0.002 % DMSO in PBS, total volume 200 μL). Following intraperitoneal administration, each rat was returned to its box and exposed to 27°C for 2 h (10:00–12:00) during the light phase, as per our previous study [10].

T_b_ and Act were measured using a built-in data logger, and T_tail_ was monitored using infrared thermography (Thermo GEAR G100; Nippon Avionics Co., Ltd., Yokohama, Japan) at 1-min intervals. An infrared thermography camera was placed 90 cm above each rat. Accuracy (±2°C) was determined based on the instruction manual. T_tail_ was determined by averaging temperature measurements recorded at two sites located one-third of the tail length from both the tip and root, based on the thermograms. When tail-hiding behavior was observed, the missing T_tail_ value was replaced with the previous time point value, as described in our previous study [24].

Tail-hiding behavior was monitored by the tail position using the same infrared thermography. The total duration of tail-hiding behavior was analyzed from 30 to 120 min after exposure to the tested chamber temperatures by summing the durations of the individual tail-hiding episodes [11], [18], [24]. We previously reported tail-hiding behavior in rats, characterized by the placement of the tail underneath the trunk, as the tail functions as an effector organ for thermoregulation. Behavior was counted when the rat hid its entire tail under its body, and this natural thermoregulatory behavior helped inhibit heat dissipation during cold exposure [25]. This duration increases with decreasing environmental temperature and has been reported to occur even at thermoneutral ambient temperatures (27°C) [11], [18], [24].

After the measurements, the rats were euthanized by intraperitoneal injection of an overdose of secobarbital sodium (15 mg/100 g body weight; Nichi-Iko Pharmaceutical Co. Ltd., Toyama, Japan). A 2-mL blood sample was collected from the left ventricular cavity to assess plasma T3 and T4 levels.

### Metabolic parameter measurements

We measured the metabolic parameters in ovariectomized rats to investigate the effects of TREK agonist treatment under different E_2_ conditions on thermogenesis. A separate cohort of operated rats formed four experimental groups: Vehicle/E_2_(–), Vehicle/E_2_(+), TREK agonist/E_2_(–), and TREK agonist/E_2_(+), as described above. At 18:00 on the day before the experiment (6 d after surgery), each rat was placed in a metabolic measurement box (21 × 36 × 22 cm; ARCO SYSTEM inc., Kashiwa, Japan) with an airflow of 1000 mL/min, and the box with the rat was moved to a climatic chamber (Program Incubator IN804, Yamato Scientific) maintained at 27°C. Oxygen consumption (VO_2_) and carbon dioxide output (VCO_2_) were measured immediately using an expired gas metabolic analyzer (Cpex-1; Inter Reha Co., Ltd, Tokyo, Japan) with a custom-ordered sample controller and software (ARCO SYSTEM Inc.) at 1-min intervals. T_b_ and Act were measured at 08:00 on the experimental day (7 d after surgery). Data collected between 9:30 and 10:00 were used as the baseline values. At 10:00, each rat was administered a vehicle or TREK agonist following the same protocol described for heat loss measurements. T_tail_ and heat loss index could not be measured because of the physical properties of the metabolic measurement box, which prevented accurate thermographic measurements through its acrylic board. After the measurements, the rats were euthanized, and blood samples were collected following the same protocol described for the heat loss measurements to assess plasma T3 and T4 levels. The bilateral dorsal root ganglia (DRGs) were dissected from all accessible spinal levels, as technically possible, for subsequent mRNA expression analysis.

### Distribution of animals for *In Vivo* studies

The total number of rats (n = 68) was divided into two groups: heat loss and thermoregulatory behavior measurements (n = 36) and metabolic parameter measurements (n = 32). T_b_ and Act data were analyzed for all 68 rats using both measurement protocols. For heat loss and thermoregulatory behavior measurements, 36 rats were used for T_tail_, heat loss index, and tail-hiding behavior duration analysis. For metabolic parameter measurements, 32 rats were used for VO_2_, VCO_2_, and mRNA expression analysis. T3 and T4 analyses were conducted on 52 and 51 rats, respectively, using rats from the heat loss and thermoregulatory behavior measurement group (n = 36) and the metabolic parameter measurement group (n = 32). The distribution of rats among the four experimental groups (Vehicle/E_2_(–), Vehicle/E_2_(+), TREK agonist/E_2_(–), and TREK agonist/E_2_(+)) is detailed in the Results section.

### Plasma thyroid hormone analysis

Plasma thyroid hormone levels were measured to evaluate thermogenesis [26]. Blood was centrifuged at 4°C, and plasma was stored at −80°C until analysis. The plasma levels of thyroid hormones T3 and T4 (Lumipulse Presto® T4; FUJIREBIO Inc., Tokyo, Japan; fully automatic CLEIA system Lumipulse L2400; FUJIREBIO Inc., Tokyo, Japan) were determined at SRL Inc. (Tokyo, Japan). All measurements were performed according to the manufacturer's instructions. T3 and T4 analyses were performed on 52 and 51 rats, respectively.

### mRNA expression analysis

Total RNA was extracted from the DRGs using the RNeasy® Plus Universal Mini Kit (QIAGEN, Tokyo, Japan) following the manufacturer’s protocol. The total RNA concentration in the eluate was determined by measuring the absorbance at 260 and 280 nm (NanoDrop One Spectrophotometer; Thermo Scientific, Wilmington, DE, USA). cDNA was then synthesized using the PrimeScript™ RT Master Mix (Takara Bio Inc., Kusatsu, Japan). RT-qPCR was performed using an RT-PCR kit (TB Green® Premix Ex Taq™, Takara Bio Inc.).

This study measured the mRNA levels of the three *Trek* subtypes of interest. Based on the heatmap from bulk RNA-seq of the DRG (Figure S1-1), group differences were observed in the transient receptor potential channel family genes, including *Trpv1*, *Trpv2*, *Trpv3*, and *Trpm8*. Therefore, we measured the mRNA expression of these genes. Additionally, the upregulated biological pathways after TREK agonist administration, identified by Metascape enrichment analysis (Figure S1-2), showed that GO:009266, which included the *Vgf*, *Ier5*, *Cdkn1a*, and *Nos1* genes related to the response to temperature stimulus. Therefore, the expression levels of these genes were analyzed by RT-PCR.

Electrophysiological studies on HEK293 cells expressing TREK1 have shown that estrogen promotes the activation of TREK1 via GPER1 [16]. We hypothesized that the expression of estrogen receptors influences the facilitation of TREK channel activation. Thus, we measured the mRNA expression of *Esr1*, *Esr2*, and *Gper1*.

*Gapdh* was used as a reference, as described in a previous report that determined the mRNA levels of *Trek1*, *Trek2*, *Traak* [27], *Trpv1*, *Trpv2*, *Trpv3* [28], *Trpm8* [29], *Vgf* [30], *Ier5* [31], *Cdkn1a* [32], *Nos1* [33], *Esr1*, *Esr2,* and *Gper1* [34]. Amplification was performed using StepOne Software v2.3 (Applied Biosystems, Foster City, CA, USA). The denaturation protocol consisted of 95°C for 30 s, 95°C for 5 s, and 64°C for 30 s for 40 cycles. mRNA quantities were calculated using the comparative Ct method assuming similar primer efficiencies.

### Slow ventral root potential (sVRP) measurements *in vitro*

*In vitro* sVRP measurements were performed to establish the fundamental neurophysiological effects of TREK channel activation on sensory signal transmission, thus providing a mechanistic basis for understanding our *in vivo* findings. It should be noted that these specific spinal cord preparations are technically feasible using only neonatal animals because the viability and accessibility of the spinal cord tissue necessary for electrophysiological recordings cannot be maintained in adult specimens. Therefore, while examining the E_2_ effects in this preparation would be valuable, the technical impossibility of performing these measurements in adult ovariectomized rats with or without E_2_ supplementation necessitated our focus on establishing the direct effects of TREK activation on sensory transmission as an essential baseline component of the peripheral mechanisms involved in thermoregulation.

Spinal cord preparations (second to sixth thoracic and first to fifth lumbar spinal cord segments) were dissected from 0- to 4-day-old Wistar rats (of either sex) that were deeply anesthetized with isoflurane as previously described [35], [36]. The preparations were placed in a 2 mL experimental chamber and continuously superfused with artificial cerebrospinal fluid (ACSF) [37] containing the following (mM): 124 NaCl, 5.0 KCl, 1.24 KH2PO4, 2.4 CaCl_2_, 1.3 MgCl_2_, 26 NaHCO_3_, and 30 glucose, equilibrated with 95 % O_2_ and 5 % CO_2_, pH 7.4, at 25–27°C.

To examine whether TREK channels modulate sensory signal transmission during cold-mimicking stimulation, sVRP was induced in the ventral roots of the fourth thoracic or fourth lumbar cord by electrical stimulation (5–20 V, 100 μs square pulse, every 30 s) applied to the dorsal roots at the same level via a glass suction electrode. The evoked responses were recorded using the same type of electrode and amplified through a 0.5 Hz high-pass filter of an AC amplifier (AB-651J, Nihon Kohden, Tokyo, Japan). The stimulus intensity was adjusted to produce approximately one-third of the maximum response, which was determined by gradually increasing the stimulus intensity until the maximum response was achieved.

The effects of TREK agonists were examined in thoracic (n = 4) and lumbar (n = 6) cord preparations from seven newborn rats. After setting up the recording conditions, the preparations were stabilized with ACSF for approximately 15 min until the spinal reflex stabilized. The peak amplitude of each sVRP was measured at 30-s intervals throughout the experimental period using LabChart 7 Pro software (ADInstruments, Castle Hill, Australia). For each time point of interest (control and during drug application), six consecutive sVRP recordings taken at 30-s intervals were averaged to obtain a representative value. Ostruthin (500 µM, initially dissolved in DMSO to a final concentration of 0.1 % and then in ACSF) was administered by bath application for 15 min to evaluate its effects on sensory signal transmission, followed by a 15-min washout period with normal ACSF.

For time-control experiments, six lumbar spinal cord preparations dissected from six newborn rats were superfused with ACSF without ostruthin for 30 min. The peak amplitude of each sVRP was measured at 30-s intervals, and six consecutive recordings were averaged at both 15 and 30 min to obtain representative values. Data were stored on a computer for offline analysis.

### Statistics

Data are presented as the mean ± standard deviation. Values for T_b_, Act, T_tail_, VO_2_, and VCO_2_ were averaged at baseline and at 0–120 min time points. Baseline values were calculated using the data collected between 09:30 and 10:00 h before the injection. One-way ANOVA was performed to examine the statistical differences in baseline values before intraperitoneal injection among the four experimental groups: Vehicle/E_2_(−), Vehicle/E_2_(+), TREK agonist/E_2_(−), and TREK agonist/E_2_(+). A two-way analysis of variance (ANOVA) was performed to examine the main effects and interactions of E_2_ status [(−) and (+)] and treatment (vehicle and TREK agonist) on T_b_, Act, T_tail_, VO_2,_ VCO_2_, the duration of tail-hiding behavior during the experiment, and on post-experimental measurements of thyroid hormones (T3 and T4), and mRNA levels of all genes in the DRG.

As this was an exploratory study designed to generate hypotheses for future research, we prioritized detecting potential effects over strict control of the family-wise error rate. Multiple independent two-tailed t-tests were performed to examine between-group differences, with the understanding that significant results should be interpreted as preliminary evidence requiring validation in subsequent confirmatory studies. For the sVRP experiments, statistical differences in peak amplitudes between the control and drug-treated conditions were assessed using a paired Student's *t*-test. Statistical analyses were performed using SPSS Statistics 30 software (IBM Corp., Armonk, NY, USA). Statistical significance was set at P < 0.05.

## Results

### Body temperature (T_b_), Tail skin temperature (T_tail_), and locomotor activity (Act)

**Fig. 1:**
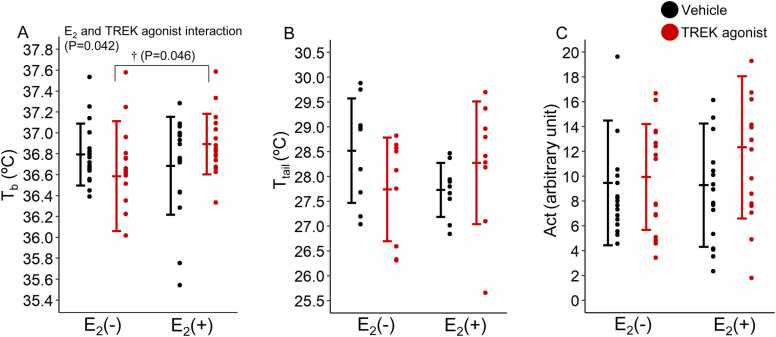
Body temperature (T_b_), tail skin temperature (T_tail_), and activity (Act) (A, B, and C, respectively). Values are presented as the mean ± standard deviation (T_b_ and Act, n = 17/group; T_tail_, n = 9/group). Differences in T_b_, Act, and T_tail_ between E_2_(−), E_2_(+), vehicle, and TREK agonist were assessed using two-way analysis of variance (ANOVA). Two-tailed t-tests were performed to compare groups where significant interactions were found: †TREK agonist/E_2_(−) vs. TREK agonist/E_2_(+). Statistical significance was set at P < 0.05. E_2_, estrogen; TREK, TWIK-related potassium channels.

Fig. 1A shows the mean T_b_ for 120 min after administration of either vehicle or TREK agonist in four experimental groups: Vehicle/E_2_(−), Vehicle/E_2_(+), TREK agonist/E_2_(−), and TREK agonist/E_2_(+). Two-way ANOVA revealed a significant interaction between the effects of E_2_ and TREK agonist [F(1,64)= 4.286, P = 0.042], with no significant main effects [E_2_: F(1,64)= 0.966, P = 0.329; TREK agonist: F(1,64)= 0.000, P = 0.985]. T_b_ in the TREK agonist/E_2_(+) group was significantly higher than that in the TREK agonist/E_2_(–) group (P = 0.046). The significant interaction between E_2_ and the TREK agonist (P = 0.042) confirmed that the effect of the TREK agonist on T_b_ was dependent on E_2_ status, with the TREK agonist producing significant T_b_ elevation specifically in the presence of E_2_. Although the observed differences between the groups (0.1°C–0.2°C) were within the range of the device accuracy (±0.5°C), the statistical significance was determined based on multiple animals per group (n = 17), which increases the statistical power and reliability of our findings. Baseline T_b_ values in all four experimental groups were comparable: [Vehicle/E_2_(–), n = 17, 36.9 ± 0.5°C; Vehicle/E_2_(+), n = 17; 36.8 ± 0.4°C, TREK agonist/E_2_(–), n = 17, 36.7 ± 0.4°C; TREK agonist/E_2_(+), n = 17, 36.9 ± 0.4°C] [P = 0.324 (one-way ANOVA)].

Fig. 1B shows the mean T_tail_ for 120 min after administration of either vehicle or TREK agonist. Two-way ANOVA revealed no significant main effects of E_2_ [F(1,32)= 0.147, P = 0.704], TREK agonist [F(1,32)= 3.936, P = 0.056], or their interaction [F(1,32)= 0.123, P = 0.728]. These results indicated that neither the TREK agonist nor E_2_ affected T_tail_. Baseline T_tail_ values were comparable across all groups: [Vehicle/ E_2_ (–), n = 9, 28.4 ± 1.5°C; Vehicle/E_2_(+), n = 9, 28.6 ± 1.7°C; TREK agonist/E_2_(–), n = 9, 27.8 ± 0.8°C; TREK agonist/E_2_(+), n = 9, 27.7 ± 0.4°C] [P = 0.235 (one-way ANOVA)].

Fig. 1C shows the mean in Act (measured using a triaxial accelerometer) for 120 min after administration of either vehicle or TREK agonist. Two-way ANOVA revealed no significant main effects of E_2_ [F(1,64)= 0.816, P = 0.370], TREK agonists [F(1,64)= 1.109, P = 0.296], or the interaction between these factors [F(1,64)= 2.083, P = 0.154], indicating that neither E_2_ nor TREK agonists affected physical activity levels. Baseline Act values were comparable across groups: [Vehicle/E_2_(–), n = 17, 4 ± 6; Vehicle/E_2_(+), n = 17, 4 ± 5; TREK agonist/E_2_(–), n = 17, 5 ± 8; TREK agonist/E_2_(+), n = 17, 6 ± 7] [P = 0.739 (one-way ANOVA)].

### Metabolic parameters

**Fig. 2:**
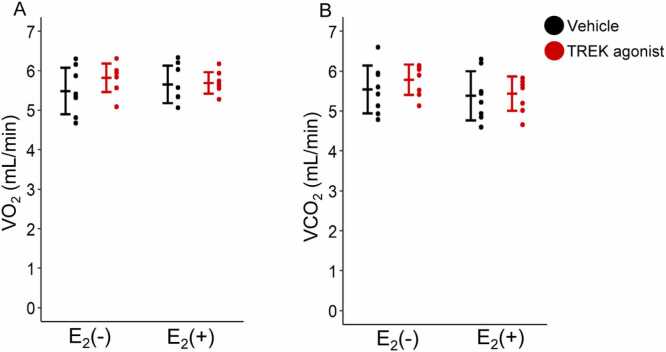
VO_2_ and VCO_2_ (A and B). Values are presented as mean ± standard deviation (VO_2_ and VCO_2_; n = 8 for each group). Differences in VO_2_ and VCO_2_ between E_2_(−), E_2_(+), vehicle, and TREK agonist were assessed using two-way analysis of variance (ANOVA). Statistical significance was set at P < 0.05. VO_2_, oxygen consumption; VCO_2_, carbon dioxide output; E_2_, estrogen; TREK, TWIK-related potassium channels.

Figs. 2A and 2B show the mean VO_2_ and VCO_2_ for 120 min after administration of either vehicle or TREK agonist. A two-way ANOVA revealed no significant effects [E_2_: F(1,28)= 0.014, P = 0.908; TREK agonist: F(1,28)= 1.411, P = 0.245; interaction: F(1,28)= 0.919, P = 0.346]. For VCO_2_, two-way ANOVA revealed no significant effects [E_2_: F(1,28)= 1.941, P = 0.175; TREK agonist: F(1,28)= 0.671, P = 0.420; interaction: F(1,28)= 0.259, P = 0.615]. These results indicated that neither the TREK agonist nor E_2_ affected metabolic parameters. Baseline values for VO_2_ and VCO_2_ in all four groups were comparable: [Vehicle/E_2_(–), n = 8; Vehicle/E_2_(+), n = 8; TREK agonist/E_2_(–), n = 8; TREK agonist/E_2_(+), n = 8] of VO_2_ (5.6 ± 1.0 mL/min; 5.3 ± 0.6 mL/min; 5.8 ± 0.5 mL/min; 5.5 ± 0.6 mL/min) and VCO_2_ (5.7 ± 0.8 mL/min; 5.2 ± 0.7 mL/min; 5.9 ± 0.6 mL/min; 5.3 ± 0.7 mL/min) [P = 0.632 and P = 0.215, respectively (one-way ANOVA)].

### Tail-hiding behavior

**Fig. 3:**
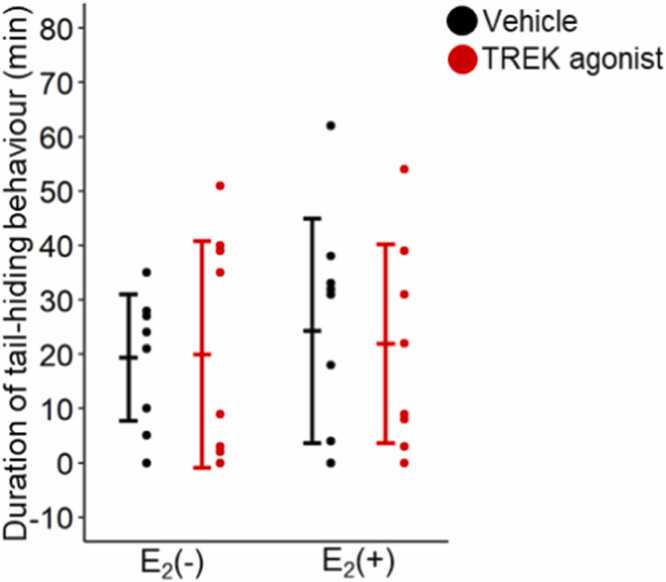
Duration of tail-hiding behavior. Values are presented as mean ± standard deviation (n = 9 for each group). All data points for the groups during the duration of tail-hiding behavior were plotted. Differences in the duration of tail-hiding behavior between the E_2_(−), E_2_(+), vehicle, and TREK agonist were assessed using two-way analysis of variance (ANOVA). Statistical significance was set at P < 0.05. E_2_, estrogen; TREK, TWIK-related potassium channels.

Fig. 3 shows the duration of the tail-hiding behavior. All four groups [Vehicle/E_2_(–), n = 9; Vehicle/E_2_(+), n = 9; TREK agonist/E_2_(–), n = 9; and TREK agonist/E_2_(+), n = 9] were included. The analysis revealed no significant main effects or interactions (TREK agonist: F(1,32)= 0.021, P = 0.885; E_2_: F(1,32)= 0.320, P = 0.576; interaction: F(1,32)= 0.056, P = 0.814 [two-way ANOVA]), indicating that neither the E_2_ status nor the TREK agonist affected tail-hiding behavior.

### Plasma thyroid hormones

**Fig. 4:**
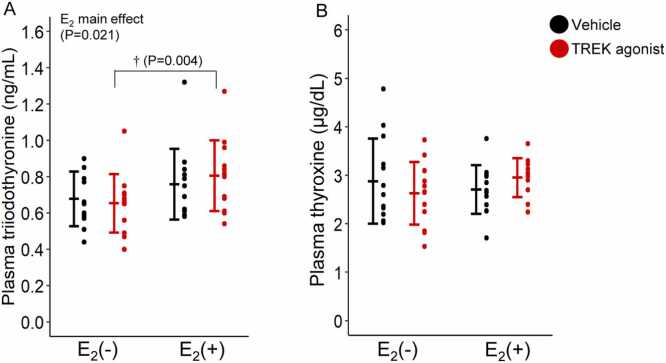
Plasma levels of triiodothyronine and thyroxine (A and B) concentrations. Values are presented as mean ± standard deviation (triiodothyronine and thyroxine, n = 13 for each group). All data points for each group are plotted. Differences in the plasma between the E_2_(−), E_2_(+), vehicle, and TREK agonist were assessed using two-way analysis of variance (ANOVA). Two-tailed t-tests were performed to compare groups where significant interactions were found:†TREK agonist/E_2_(−) vs. TREK agonist/E_2_(+). Statistical significance was set at P < 0.05. E_2_, estrogen; TREK, TWIK-related potassium channels.

Fig. 4 shows the plasma concentrations of thyroid hormones (T3 and T4; Figs. 4A and 4B). The sample sizes for each group were as follows: for T3 (Vehicle/E₂(–), n = 13; Vehicle/E₂(+), n = 13; TREK agonist/E₂(–), n = 13; TREK agonist/E₂(+), n = 13), and T4 (Vehicle/E₂(–), n = 13; Vehicle/E₂(+), n = 13; TREK agonist/E₂(–), n = 13; TREK agonist/E₂(+), n = 12). For thyroid hormones, a significant main effect of E_2_ [F(1,48)= 5.677, P = 0.021] was observed on T3 concentration, with no TREK agonist effect (F(1,48)= 0.052, P = 0.821) or interaction (F(1,48)= 0.535, P = 0.468). The T3 concentration in the TREK agonist/E_2_(+) group was significantly higher (P = 0.004) than that in the TREK agonist/E_2_(–) group. No significant effects were observed for T4 (TREK agonist: F(1,47)= 0.025, P = 0.876; E_2_: F(1,47)= 0.363, P = 0.550; interaction: F(1,47)= 2.409, P = 0.127). These results indicated that E₂ increased T3 levels regardless of the presence of the TREK agonist.

### mRNA expression analysis

**Fig. 5:**
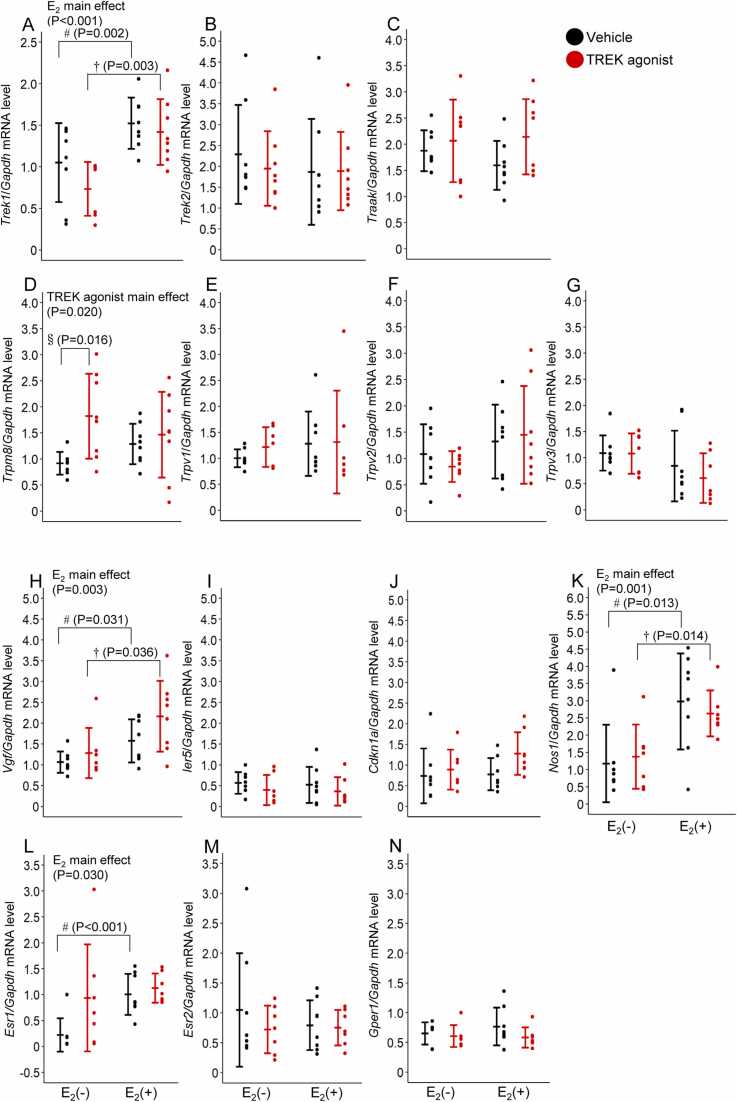
*Trek1, Trek2, Traak, Trpm8, Trpv1, Trpv2, Trpv3, Vgf, Ier5, Cdkn1a, Nos1, Esr1, Esr2*, and *Gper1* (A-N) mRNA expression in the DRG. Values are presented as mean ± standard deviation (n = 8 for each group). All data points for each group are plotted. Differences in mRNA levels of all genes in the DRG between the E_2_(−), E_2_(+), vehicle, and TREK agonist were assessed using two-way analysis of variance (ANOVA). Two-tailed t-tests were performed to compare groups where significant interaction and main effects were found: § Vehicle/E_2_(−) vs. TREK agonist/E_2_(−); # Vehicle/E_2_(−) vs. Vehicle/E_2_(+); †TREK agonist/E_2_(−) vs. TREK agonist/E_2_(+). Statistical significance was set at P < 0.05. DRG, dorsal root ganglia; E_2_, estrogen; TREK, TWIK-related potassium channels.

Fig. 5 shows the mRNA expression levels of *Trek1*, *Trek2*, *Traak*, *Trpm8*, *Trpv1*, *Trpv2*, *Trpv3*, *Vgf*, *Ier5*, *Cdkn1a*, *Nos1*, *Esr1*, *Esr2*, and *Gper1* (Fig. 4A-4N). All four groups [Vehicle/E_2_(–), n = 8; Vehicle/E_2_(+), n = 8; TREK agonist/E_2_(–), n = 8; and TREK agonist/E_2_(+), n = 8] were included. Two-way ANOVA revealed significant main effects of E_2_ on *Trek1, Vgf, Nos1*, and *Esr1* mRNA expression, whereas the TREK agonist significantly affected *Trpm8* mRNA (Table 1). Follow-up comparisons using independent t-tests indicated that *Trek1, Vgf,* and *Nos1* mRNA levels were higher in the E_2_(+) groups, *Esr1* was higher in the E_2_(+)/Vehicle group, and *Trpm8* was higher in the TREK agonist/E_2_(−) group compared to their respective controls (Fig. 4A, D, H, K, and L). No significant effects were observed in the remaining genes examined (Table 1).

**Table 1:** Two-way ANOVA results for mRNA expression levels in the dorsal root ganglia.

**Gene**	**E2 Effect**	**TREK Agonist Effect**	**Interaction**	**Significant Differences**
*Trek1*	F(1,27)= 17.553, P < 0.001*	F(1,27)= 2.338, P = 0.138	F(1,27)= 0.596, P = 0.447	Higher in E2(+) groups (Vehicle: P = 0.002, TREK agonist: P = 0.003)
*Trpm8*	F(1,28)= 0.001, P = 0.978	F(1,27)= 17.553, P = 0.020*	F(1,28)= 2.738, P = 0.109	Higher in TREK agonist/E2(−) group (P = 0.016)
*Vgf*	F(1,27)= 10.531, P = 0.003*	F(1,27)= 3.530, P = 0.071	F(1,27)= 0.765, P = 0.389	Higher in E2(+) groups (Vehicle: P = 0.031, TREK agonist: P = 0.036)
*Nos1*	F(1,26)= 14.996, P = 0.001*	F(1,26)= 0.036, P = 0.850	F(1,26)= 0.486, P = 0.492	Higher in E2(+) groups (Vehicle: P = 0.013, TREK agonist: P = 0.014)
*Esr1*	F(1,26)= 5.306, P = 0.030*	F(1,26)= 3.930, P = 0.058	F(1,26)= 1.974, P = 0.172	Higher in E2(+)/Vehicle group (P < 0.001)
*Trek2*	F(1,28)= 0.397, P = 0.534	F(1,28)= 0.173, P = 0.680	F(1,28)= 0.217, P = 0.645	No significant differences
*Traak*	F(1,28)= 0.223, P = 0.641	F(1,28)= 2.863, P = 0.102	F(1,28)= 0.682, P = 0.416	No significant differences
*Trpv1*	F(1,26)= 0.699, P = 0.411	F(1,26)= 0.305, P = 0.586	F(1,26)= 0.165, P = 0.688	No significant differences
*Trpv2*	F(1,27)= 3.032, P = 0.093	F(1,27)= 0.050, P = 0.825	F(1,27)= 0.565, P = 0.459	No significant differences
*Trpv3*	F(1,26)= 3.996, P = 0.056	F(1,26)= 0.462, P = 0.503	F(1,26)= 0.391, P = 0.537	No significant differences
*Ier5*	F(1,26)= 0.086, P = 0.772	F(1,26)= 1.518, P = 0.229	F(1,26)= 0.002, P = 0.969	No significant differences
*Cdkn1a*	F(1,27)= 1.329, P = 0.259	F(1,27)= 3.014, P = 0.094	F(1,27)= 0.861, P = 0.362	No significant differences
*Esr2*	F(1,26)= 0.279, P = 0.602	F(1,26)= 0.724, P = 0.403	F(1,26)= 0.437, P = 0.515	No significant differences
*Gper1*	F(1,25)= 0.278, P = 0.603	F(1,25)= 1.843, P = 0.187	F(1,25)= 0.685, P = 0.416	No significant differences

Asterisks indicate statistical significance: *P < 0.05. Statistical analysis was performed using two-way analysis of variance (ANOVA). Multiple independent two-tailed t-tests were performed to examine between-group differences. DRG, dorsal root ganglia.

### Slow ventral root potential measurements *in vitro*

**Fig. 6:**
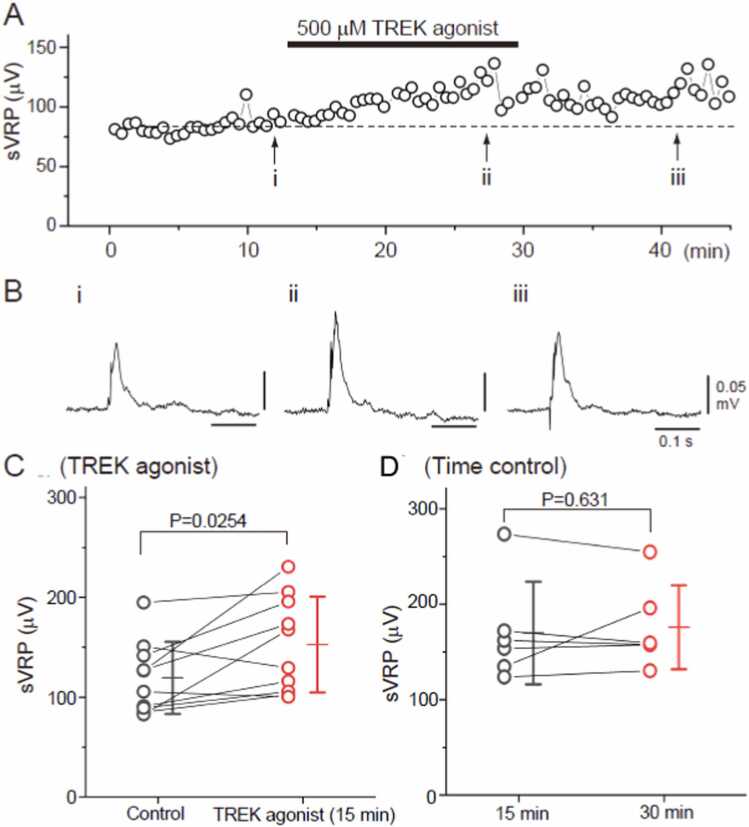
Typical example of the effect of ostruthin on sVRP in the spinal cord of newborn rats. Time course of the changes in the peak amplitude of sVRP in response to the application of 500 µM ostruthin (A). Examples of the reflex responses in the control (B-i), at 15 min after the administration of the TREK agonist (B-ii), and after 15 min of washout (B-iii). Bi-iii corresponds to i-iii in panel A. Data plots of the sVRP amplitude from 10 preparations at control (black) and the TREK agonist application for 15 min (red) (C). The bars on the right side of the individual datasets denote the mean ± standard deviation. * P < 0.05 by paired *t*-test. Data plots of the sVRP amplitude from five preparations in time control; at control (black) and 15 min after the control data were acquired (red) (D). The bars on the right side of the individual datasets denote the mean ± standard deviation. TREK, TWIK-related potassium channels; sVRP, slow ventral root potential.

Fig. 6 shows the effect of ostruthin (500 µM) on the sVRP in newborn rat spinal cord preparations, including four thoracic and six lumbar preparations from seven animals. After setting up each preparation, ACSF was perfused for approximately 15 min to establish baseline conditions. Control sVRP measurements were taken immediately before administering ostruthin. At each measurement point, six sVRP recordings were taken at 30-s intervals (approximately 150 s), with the average used as the representative value.

A representative time course of the sVRP peak amplitude is shown in Fig. 6A, with each time point representing an individual sVRP measurement recorded at 30-s intervals during the experiment. Examples of reflex responses are presented in Fig. 6B: before ostruthin administration (control, B-i), at the end of the 15-min ostruthin perfusion (B-ii), and 15 min after washout (B-iii). The traces shown in panels B-i to B-iii correspond to the measurements taken at time points i–iii marked in panel A.

The average peak amplitude of the sVRP increased from 119.4 ± 36.0 µV in the control conditions to 152.6 ± 47.9 µV after 15 min of ostruthin perfusion (P = 0.0254, two-tailed paired *t*-test). A statistical summary of the ten preparations is shown in Fig. 6C.

Time-control experiments were conducted using six lumbar cord preparations from six newborn rats to confirm that the observed changes were not time-dependent. ACSF was perfused continuously for more than 30 min. The sVRP measurements were taken at 15 min (corresponding to the pre-ostruthin time point in the test experiments) and 30 min (corresponding to the end of ostruthin perfusion in the test experiments). For each time point, six measurements were taken at 30-s intervals and averaged. No significant change in the peak amplitude of the sVRP was observed between these time points (170.0 ± 53.8 µV at 15 min vs 176.0 ± 44.0 µV at 30 min; P = 0.631, two-tailed paired *t*-test). A statistical summary of time control is shown in Fig. 6D.

## Discussion

This study investigated the interaction between the E_2_ and TREK channels in thermoregulation using ovariectomized rats as a menopausal model. Our findings revealed novel mechanisms by which E_2_ modulates TREK channel-mediated temperature regulation, with important implications for understanding thermoregulatory symptoms in menopausal women.

The most significant finding was the TREK agonist and E_2_ interaction in T_b_. Statistical analysis revealed a significantly higher T_b_ in the E_2_(+)/TREK agonist group than in the E_2_(–)/TREK agonist group. This indicates that the TREK agonist induces T_b_ elevation, specifically in the presence of E_2._ These results supported our hypothesis that E_2_ facilitates TREK-mediated thermoregulation.

T3 levels showed a main effect of E_2_, with significantly higher levels observed in the E_2_(+) group after the TREK agonist administration. This suggests that the T3-mediated heat production may underlie the T_b_ elevation observed in the presence of E_2_. Interestingly, we found no significant differences among groups in VO_2_, T_tail_, or Act. These findings indicated that conventional mechanisms (whole-body oxygen consumption, peripheral vasomotor control, and activity-dependent heat production) do not explain T_b_ changes. One possible explanation is that the E_2_-TREK interaction influences localized thermogenesis in the interscapular brown adipose tissue, which plays a crucial role in non-shivering thermogenesis in rats [38] through T3-mediated peripheral pathways rather than increased sympathetic drive [39], [40], thus suggesting more complex regulatory mechanisms than previously anticipated.

Our electrophysiological experiments revealed that the TREK agonist increased the amplitude of sVRP in the sensory nerves during cold-mimicking electrical stimulation. Since TREK channel is a type of K^+^ channels, the activation of this channel typically induces membrane hyperpolarization [41]. We propose that the mild hyperpolarization by TREK channel activation might facilitate the recovery of voltage-dependent Na^+^ channels from inactivation [42], potentially shortening the refractory period and enhancing responses to consecutive stimuli. These results suggest that the TREK agonist enhances signal transmission when sensory nerves receive cold stimuli, potentially allowing for stronger conduction of sensory stimuli from the skin to the central nervous system. This enhanced signal transmission may partly contribute to our finding that the TREK agonist elevated T_b_ under E_2_ administration.

RNA-seq and RT-PCR analyses identified temperature-responsive genes modulated by E_2_, particularly *Vgf* and *Nos1*. The upregulation of *Vgf* by E_2_ may be particularly relevant, given that *Vgf* knockout mice show impaired T_b_ maintenance during cold exposure [43]. While *Nos1* is widely expressed in various tissues, including sensory nerves [44] its function-specific role in peripheral cold sensing mechanisms of the DRG remains to be elucidated and could reveal novel neuromodulatory pathways in thermoregulation.

Among the E_2_-regulated genes, the increased *Trek1* expression is particularly noteworthy. This suggests a novel mechanism by which E_2_ not only directly modulates TREK channel function as previously shown *in vitro* [16], but also enhances the system's sensitivity to TREK channel activation by increasing channel expression. This dual mechanism of direct channel modulation and increased expression may explain the robust interaction between E_2_ and TREK agonists.

TREK agonist treatment increased *Trpm8* expression in the DRG of E_2_(–) rats yet did not significantly alter T_b_ in this group. In contrast, E_2_(+) rats showed no changes in *Trpm8* expression following TREK agonist treatment but showed T_b_ elevation. This observation suggests that the thermoregulatory effects of TREK channel activation may involve mechanisms beyond simple changes in *Trpm8* expression and highlights the potential importance of E_2_ in this process.

In conclusion, our findings demonstrate that a TREK agonist induces T_b_ elevation specifically in the presence of E_2_, suggesting that E_2_ creates a permissive environment for TREK channel-mediated thermoregulation through molecular changes in the DRG and enhanced signal transmission in sensory nerves. These findings have important clinical implications, particularly for understanding thermoregulatory symptoms in menopause. The complex interaction between TREK channels and E_2_, followed by regulating heat production through thyroid hormones, may explain the seemingly contradictory symptoms of hot flashes and chills in menopausal women.

Our study had several limitations. First, while the observed differences between the groups were small in magnitude, our adequate sample size (n = 17 per group) provided sufficient statistical power to detect these subtle but statistically significant differences. Second, although we observed changes in T3 levels suggesting increased thermogenesis, we did not directly measure thermogenic activity. Third, our electrophysiological experiments lacked direct evidence of the proposed Na^+^ channel recovery mechanism. Fourth, our findings differ from our previous study [18], in which ostruthin increased body temperature in ovariectomized rats. In the present study, ostruthin alone did not elevate body temperature but showed significant effects only in the presence of E₂. This discrepancy suggests that TREK-mediated thermoregulation may be influenced by hormonal environment such as E₂. Finally, as our experiments were conducted at thermoneutral temperatures, the role of the E₂-TREK interaction under different thermal challenges requires further investigation.

## CRediT authorship contribution statement

**Shotaro Kamijo:** Investigation. **Yuki Uchida:** Writing – review & editing, Writing – original draft, Visualization, Validation, Supervision, Resources, Project administration, Methodology, Investigation, Funding acquisition, Formal analysis, Data curation, Conceptualization. **Masahiko Izumizaki:** Writing – review & editing, Writing – original draft, Visualization, Validation, Supervision, Investigation, Formal analysis, Data curation. **Yuri Masaoka:** Writing – review & editing, Writing – original draft, Supervision, Investigation, Data curation. **Motoyasu Honma:** Writing – review & editing, Writing – original draft, Supervision, Formal analysis, Data curation. **Keiko Ikeda:** Investigation. **Hikaru Isobe:** Writing – original draft, Validation, Investigation. **Masahiro Hosonuma:** Writing – review & editing, Writing – original draft, Supervision, Investigation, Formal analysis. **Hiroshi Onimaru:** Writing – review & editing, Writing – original draft, Visualization, Investigation. **Yuki Samejima:** Investigation.

## Consent for publication

Not applicable.

## Ethics approval and consent to participate

All experimental protocols were approved by the Animal Research Committee of Showa Medical University (Tokyo, Japan) (approval number: 03110) and conducted in accordance with institutional guidelines for animal care and use.

## Funding

This study was supported by a Japan Society for the Promotion of Science Grant-in-Aid for Scientific Research (C) No. 20K07275.

## Declaration of Competing Interest

The authors declare that they have no competing interests.

## Data Availability

Raw data were generated at Showa Medical University. Derived data supporting the findings of this study are available from the corresponding author Y.U. upon reasonable request.

## Footnotes

Appendix A Supplementary data associated with this article can be found in the online version at doi:10.1016/j.jphyss.2025.100044.
