# Supplementary figures and images for: Estradiol enhances thermoregulation induced by ostruthin, a TREK channel agonist, in ovariectomized rats
Source: J Physiol Sci. 2025 Sep 26;75(3):100044. doi: 10.1016/j.jphyss.2025.100044 (PMC12509895; doi:10.1016/j.jphyss.2025.100044)

# Supplementary figures:

## Figure S1-1

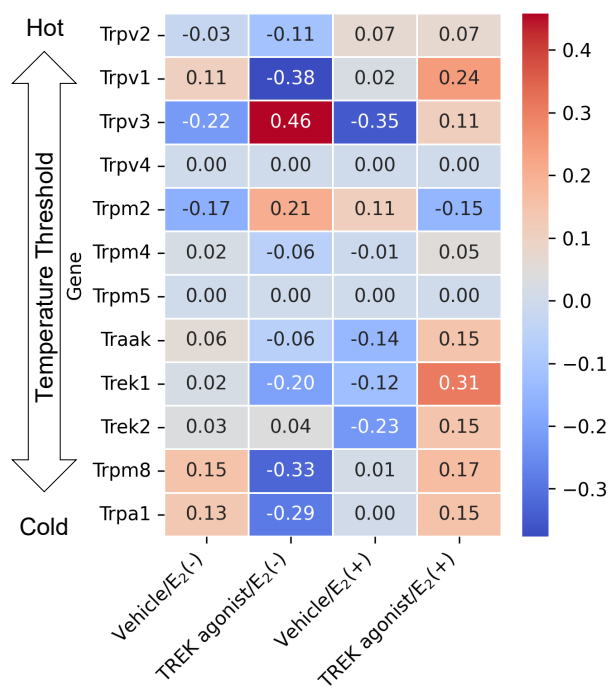

Figure S1-2

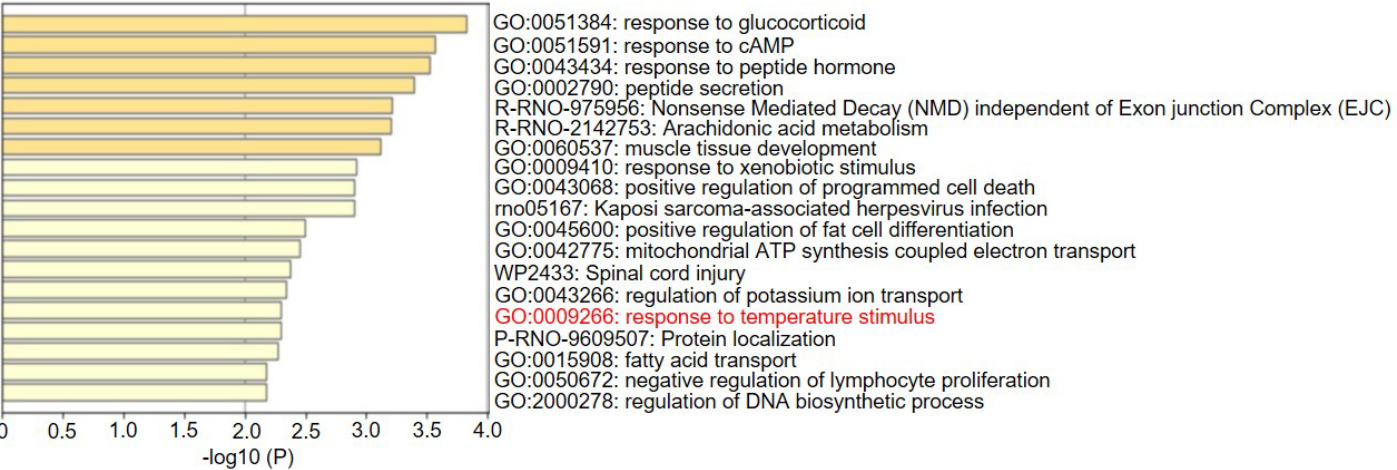

Supplement: Supplementary file 1 — Supplementary material [file mmc1.pdf]
