# Supplementary material for: Estradiol enhances thermoregulation induced by ostruthin, a TREK channel agonist, in ovariectomized rats
Source: J Physiol Sci. 2025 Sep 26;75(3):100044. doi: 10.1016/j.jphyss.2025.100044 (PMC12509895; doi:10.1016/j.jphyss.2025.100044)
Supplement: Supplementary file 2 — Supplementary material [file mmc2.docx]

**Supplementary results**

**Bulk RNA-seq**

We observed differentially expressed genes in the *Trp* and *Trek* gene families in the heatmap of bulk RNA-seq from the DRG (Figure S1-1). Group differences were observed in the expression of *Trek1*, *Trek2*, *Traak*, *Trpm8*, *Trpv1*, *Trpv2*, and *Trpv3*. Figure S1-2 shows the upregulated biological pathways after TREK agonist administration, identified by Metascape enrichment analysis. GO:009266, which included the *Vgf*, *Ier5*, *Cdkn1a*, and *Nos1* genes, was related to response to temperature stimulus.

**Supplementary Figures**

Figure S1-1. Heatmap displaying gene expressions of *Trek* and *Trp* channels in the normalized values of the bulk RNA-seq in the DRG. The values were normalized using Seurat (https://satijalab.org/seurat/) (n=1 for each group). The heatmap was created using Python. TREK, TWIK-related potassium channels; TRP, transient receptor potential channels; DRG, dorsal root ganglia.

Figure S1-2. The upregulated biological pathways after TREK agonist administration from Metascape (https://metascape.org/) enrichment analysis of the top 100 genes in the TREK agonist/E_2_(+) group were more than double those in the Vehicle/E_2_(+) group (n=1 for each group). GO:009266, which includes *Vgf, Ier5, Cdkn1a,* and *Nos1*, is the pathway of the response to temperature stimulus. The expression of these genes was evaluated by RT-PCR, as shown in Figure 5H-K. TREK, TWIK-related potassium channel.
